# Capecitabine-Associated Loss of Fingerprints: Report of Capecitabine-Induced Adermatoglyphia in Two Women with Breast Cancer and Review of Acquired Dermatoglyphic Absence in Oncology Patients Treated with Capecitabine

**DOI:** 10.7759/cureus.969

**Published:** 2017-01-09

**Authors:** Philip R Cohen

**Affiliations:** 1 Department of Dermatology, University of California, San Diego

**Keywords:** adermatoglyphia, cancer, capecitabine, carcinoma, dermatogyphic, fingerprint, foot, hand, syndrome, xeloda

## Abstract

Capecitabine, an oral 5-fluorouracil prodrug, is currently used in the treatment of metastatic colorectal carcinoma and breast cancer. Fingerprints, also referred to as dermatoglyphics and characterized by the pattern of ridges and furrows on the fingertips, are used for identification by government agencies and personal electronic devices. Two women with breast cancer who were treated with capecitabine and developed drug-associated loss of their fingerprints are described. PubMed was used to search the following terms separately and in combination: absence, adermatoglyphia, breast, cancer, capecitabine, carcinoma, colon, colorectal, dermatoglyphics, fingerprint, fluorouracil, foot, hand, loss, malignancy, nasopharyngeal, oncology, reaction, rectal, skin, syndrome, tumor, and xeloda. The papers identified were reviewed and appropriate references were evaluated. The characteristics of capecitabine-induced adermatoglyphia in 20 oncology patients are reviewed. Most of the patients received either 2000 mg/m^2 ^or 3500 mg, in divided doses, each day. Hand-foot syndrome, varying in severity from grade 1 to grade 4, always preceded the onset of fingerprint loss. The discovery of adermatoglyphia occurred as early as two weeks to as late as 3½ years after starting capecitabine. Patients were often unaware of their fingerprint loss until they experienced delays attempting to enter the United States, were unable to process government documents or obtain a driver’s license, or could not obtain access to their telephone, computer or gym which required fingerprint identification scanning. The loss of fingerprints was reversible for some of the individuals; however, several of the patients did not recover their dermatoglyphics, the functional quality of their fingerprints, or both after discontinuing the drug. The significance of capecitabine-induced adermatoglyphia will continue to increase as fingerprint identification continues to advance not only in scanning technology but also in global utilization. Therefore, it is essential that patients receiving capecitabine are aware of this potential adverse cutaneous sequellae.

## Introduction

Dermatoglyphics refers to the pattern of ridges and furrows on the digits of the hands and feet of an individual; however, the term is usually used synonymously with fingerprints [1]. Capecitabine is an oral, enzymatically-activated prodrug of 5-fluorouracil [2]. Acquired adermatoglyphia during treatment with capecitabine in two women with breast cancer is described and capecitabine-associated loss of fingerprints in oncology patients is reviewed. Informed consent was obtained from the patients for this study.

## Case presentation

### Case 1

A 57-year-old Caucasian woman presented for a total body skin check in November 2016. A melanoma in situ on her left proximal arm had been diagnosed five months earlier; an excision of the site had been performed two months ago. There was no evidence of recurrence and no palpable axillary lymph nodes.

Her past medical history was remarkable for right triple negative (estrogen receptor, progesterone receptor, and HER2) invasive ductal carcinoma of the breast diagnosed in June 2015. She received 12 weekly doses of paclitaxel followed by doxorubicin/cyclophosphamide every two weeks for four cycles. In December 2015 she had a right lumpectomy and sentinel lymph node biopsy; none of the three nodes were positive for cancer.

She completed radiation therapy in March 2016. Adjuvant capecitabine was recommended; in May 2016 she began oral therapy at a dose of 1500 mg twice daily; within one week, the dose was increased to 1650 mg twice daily for 14 days on and seven days off for eight cycles. She developed hand-foot syndrome during the initial cycle. This persisted during her treatment, but never greater than grade 1 in severity; neither discontinuation nor dose reduction of capecitabine was necessary.

Following the initial cycle of therapy, she became aware of a loss of fingerprint quality. She was no longer able to gain entrance into her fitness center that required index finger scanning for identification to access the facility. In addition, prior to starting capecitabine, she was able to open her smartphone by pressing her index finger on the screen; after her first cycle of capecitabine, the personal electronic device could not confirm her identity based on evaluation of her fingerprint and she has to enter a four-digit code to be able to use her telephone.

**Figure 1:**
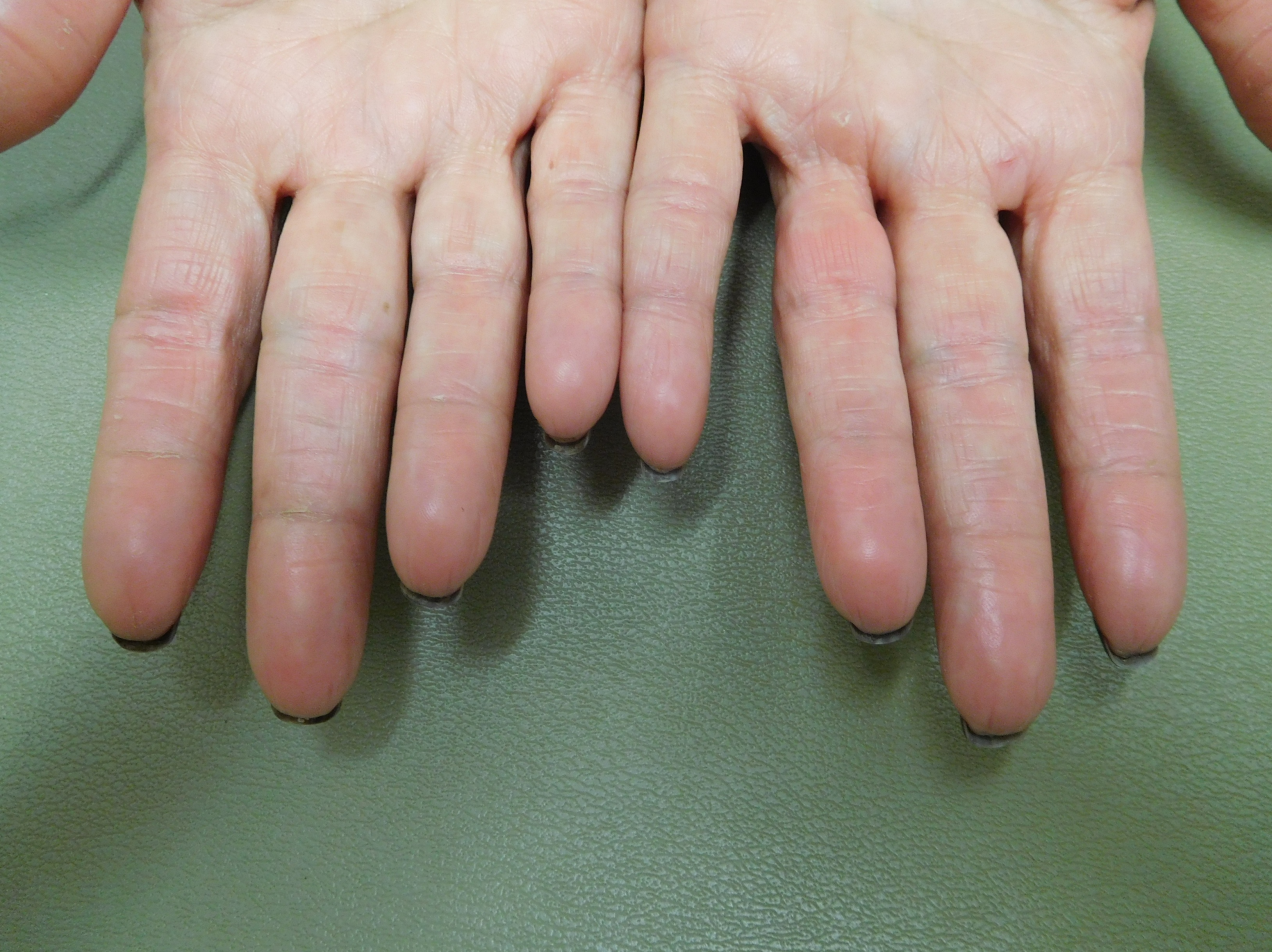
Capecitabine-associated adermatoglyphia of the fingers on both hands of a 57-year-old woman with breast cancer. After seven cycles of capecitabine (1650 mg twice daily for 14 days followed by seven days without treatment), the fingertips are red and smooth without discernable fingerprints. The functional quality of her finger dermatoglyphics was lost. She was unable to enter her fitness center that required scanning of her index finger for identification, and she was not able to open her smartphone by pressing her index finger on the screen to confirm her identity.

**Figure 2:**
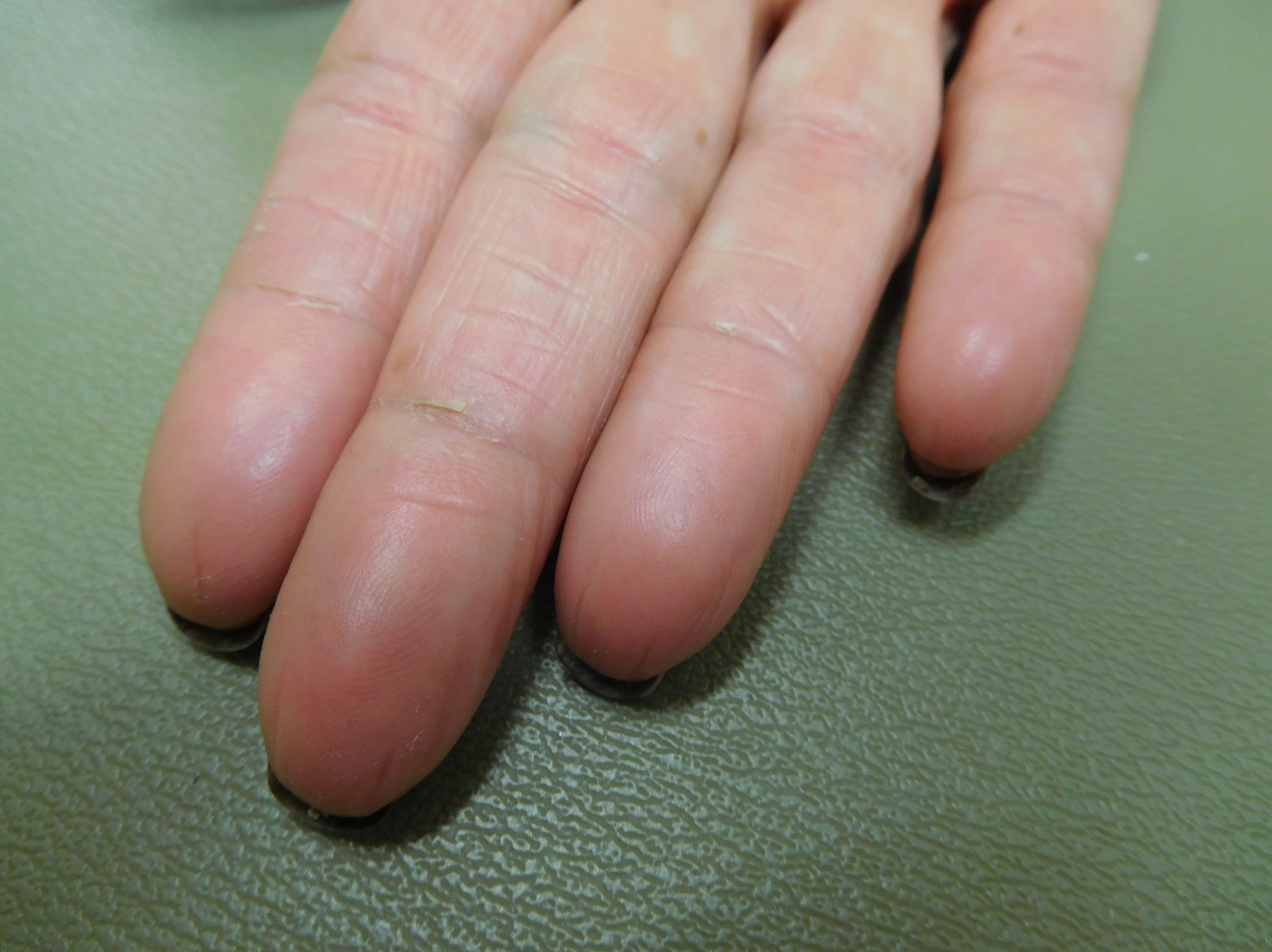
Capecitabine-associated adermatoglyphia of the fingers on the right hand of a 57-year-old woman with breast cancer. Fingerprints are absent on the erythematous smooth fingertips after seven cycles of capecitabine (1650 mg twice daily for 14 days followed by seven days without treatment); distal fissures are present.

**Figure 3:**
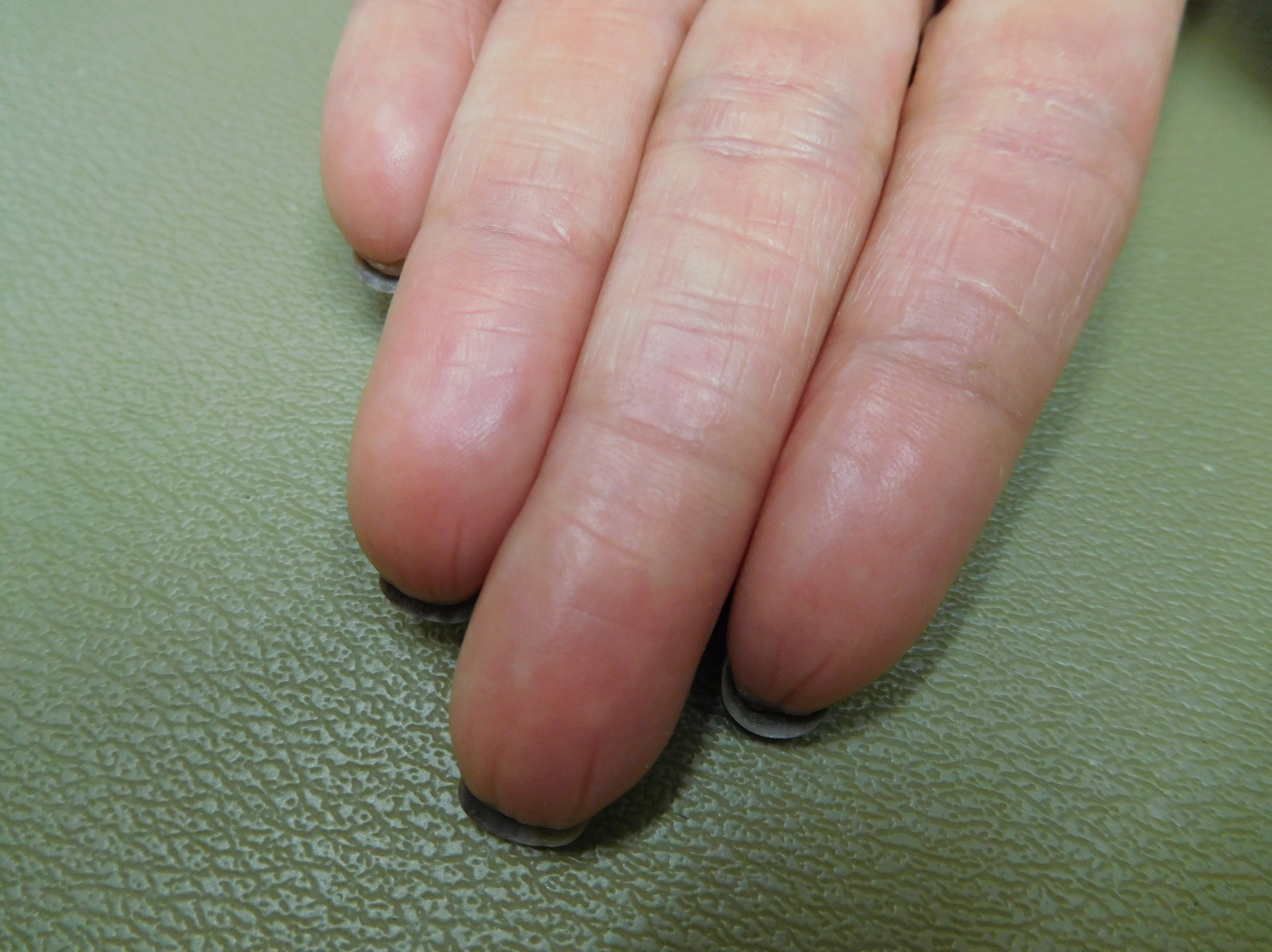
Capecitabine-associated adermatoglyphia of the fingers on the left hand of a 57-year-old woman with breast cancer. Fingerprints are absent on the erythematous smooth fingertips after seven cycles of capecitabine (1650 mg twice daily for 14 days followed by seven days without treatment); distal fissures are present.

**Figure 4:**
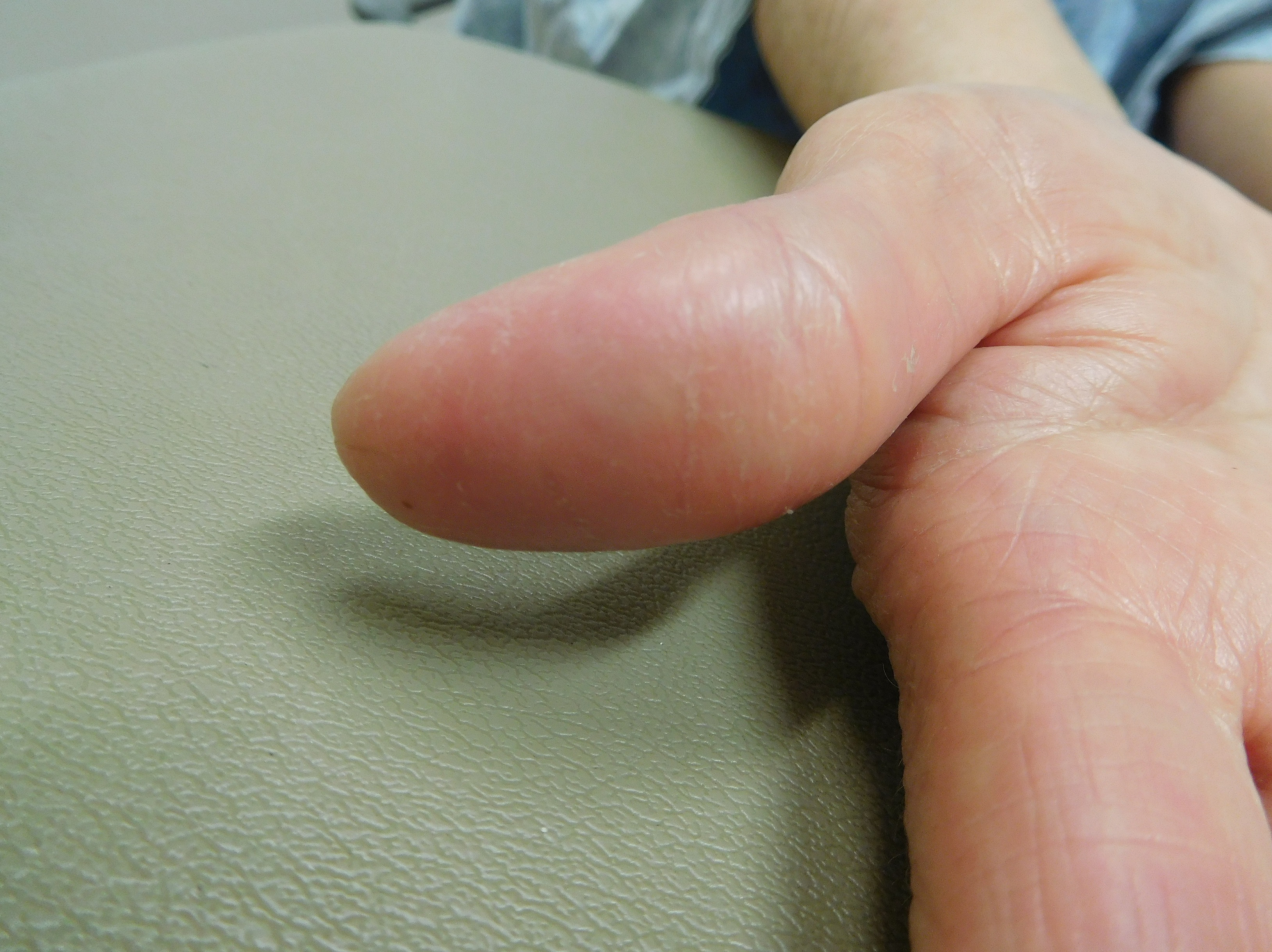
Capecitabine-induced fingerprint loss of the right thumb of a 57-year-old woman with breast cancer. A side view of the distal phalanx of the right thumb shows a red smooth skin surface with random fissures after seven cycles of capecitabine (1650 mg twice daily for 14 days followed by seven days without treatment). Fingerprints cannot readily be observed.

**Figure 5:**
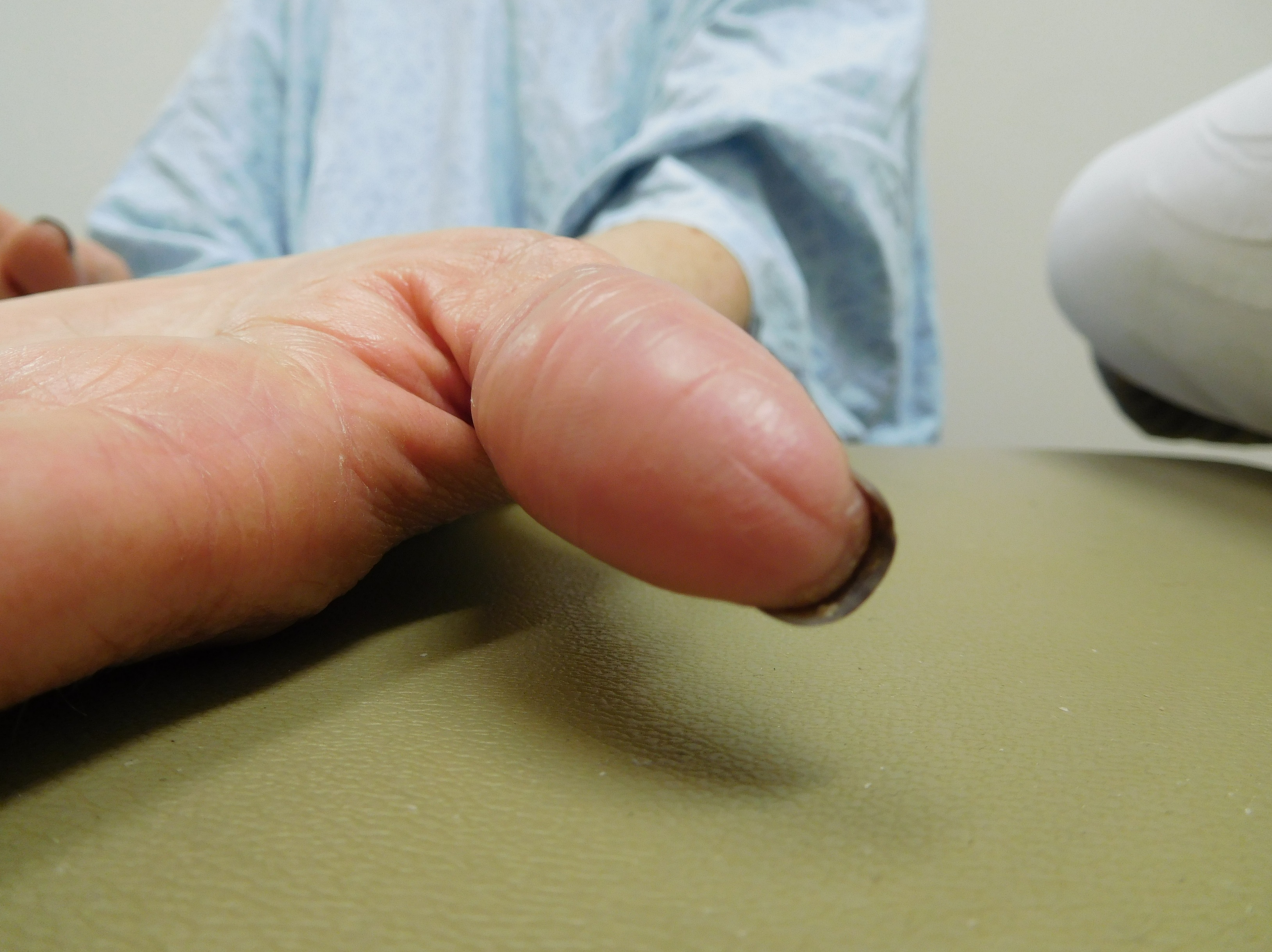
Capecitabine-induced fingerprint loss of the left thumb of a 57-year-old woman with breast cancer. A side view of the distal phalanx of the left thumb shows a red smooth skin surface with random fissures after seven cycles of capecitabine (1650 mg twice daily for 14 days followed by seven days without treatment). Fingerprints cannot readily be observed.

**Figure 6:**
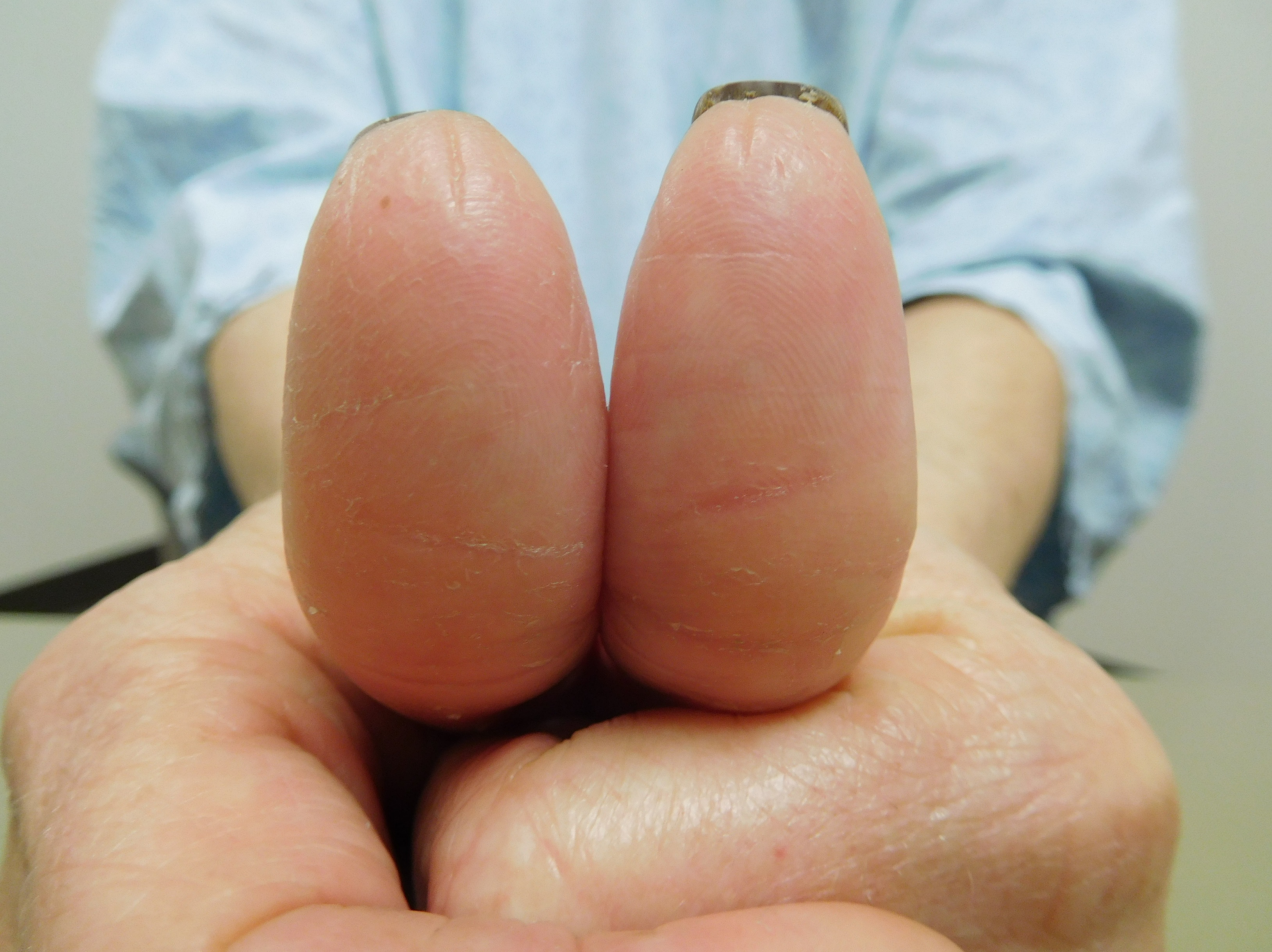
Capecitabine-induced fingerprint loss of the thumbs of a 57-year-old woman with breast cancer. After seven cycles of capecitabine (1650 mg twice daily for 14 days followed by seven days without treatment), a closer frontal view of both thumbs shows the smooth surface of the digits with mild erythema, scaling, and focal fissures. There is minimal preservation of some of the fingerprint ridges.

Examination of her hands in November 2016, just prior to her completing the final cycle of capecitabine, showed erythema of the palmar surface of her fingertips (Figures 1-3) and thumbs (Figures 4-6). A closer examination of her fingertips showed occasional distal fissures and an absence of the dermatoglyphics (Figures 2-3). A closer examination of her thumbs showed scaling, random fissures and focal preservation of only some of the fingerprint ridges (Figures 5-6). However, the loss of her fingerprints on her digits was severe with absent functional quality of her finger dermatoglyphics.

### Case 2

A 73-year-old Caucasian woman presented for a total body skin check in November 2016. A squamous cell carcinoma on her chest had been diagnosed three months earlier; an excision of the site had been performed two months ago. There was no evidence of recurrence and no palpable axillary lymph nodes.

Her past medical history was remarkable for right triple negative invasive ductal carcinoma of the breast, with metastases to three of 20 ipsilateral lymph nodes, diagnosed in 1991. She had a mastectomy with autologous latissimus dorsi flap reconstruction. She also received adjuvant chemotherapy with cyclophosphamide, methotrexate, and 5-fluorouracil.

In January 2009, she presented with a right axillary mass and lung nodules; excision of the mass showed invasive ductal carcinoma. From February 2009 to June 2009, she was treated with doxorubicin/cyclophosphamide followed by paclitaxel. The site was also treated with radiotherapy.

Follow-up evaluation in February 2012 demonstrated metastases to bone. Biopsy demonstrated triple negative adenocarcinoma. Pathology review of the right axillary mass from 2009 established a revised diagnosis of invasive, intermediate to high grade, apocrine carcinoma; subsequent genomic analysis of the tissue in November 2012 revealed an AKT E17K mutation which suggested that the mTOR inhibitor everolimus might be effective.

Capecitabine was started in April 2012 at a daily dose of 2000 mg each morning and 1500 mg each evening for one week on and one week off. Shortly after starting therapy, she developed mild hand-foot syndrome of grade 1 severity and noted that her fingertips had become smooth. She also observed that her new laptop computer would not recognize her index fingerprint to permit access. This inability of fingerprint access persisted not only for the duration of capecitabine treatment (which was stopped in October 2014) but also for more than two additional years (to her examination in November 2016).

New bone metastases were discovered in October 2014. Biopsy showed triple negative adenocarcinoma, and subsequent molecular profiling of the bone lesion in March 2015 showed AKT E17K and DNMT3A mutations; PDL-1 testing was negative. Capecitabine was discontinued and bicalutamide was started. Therapy was changed to exemestane and everolimus in March 2015.

She developed malignant pleural effusions. In August 2015, therapy was changed to eribulin. She developed new bone metastases in September 2016 that were treated with radiation therapy; eribulin was discontinued and fluoxymesterone was started.

**Figure 7:**
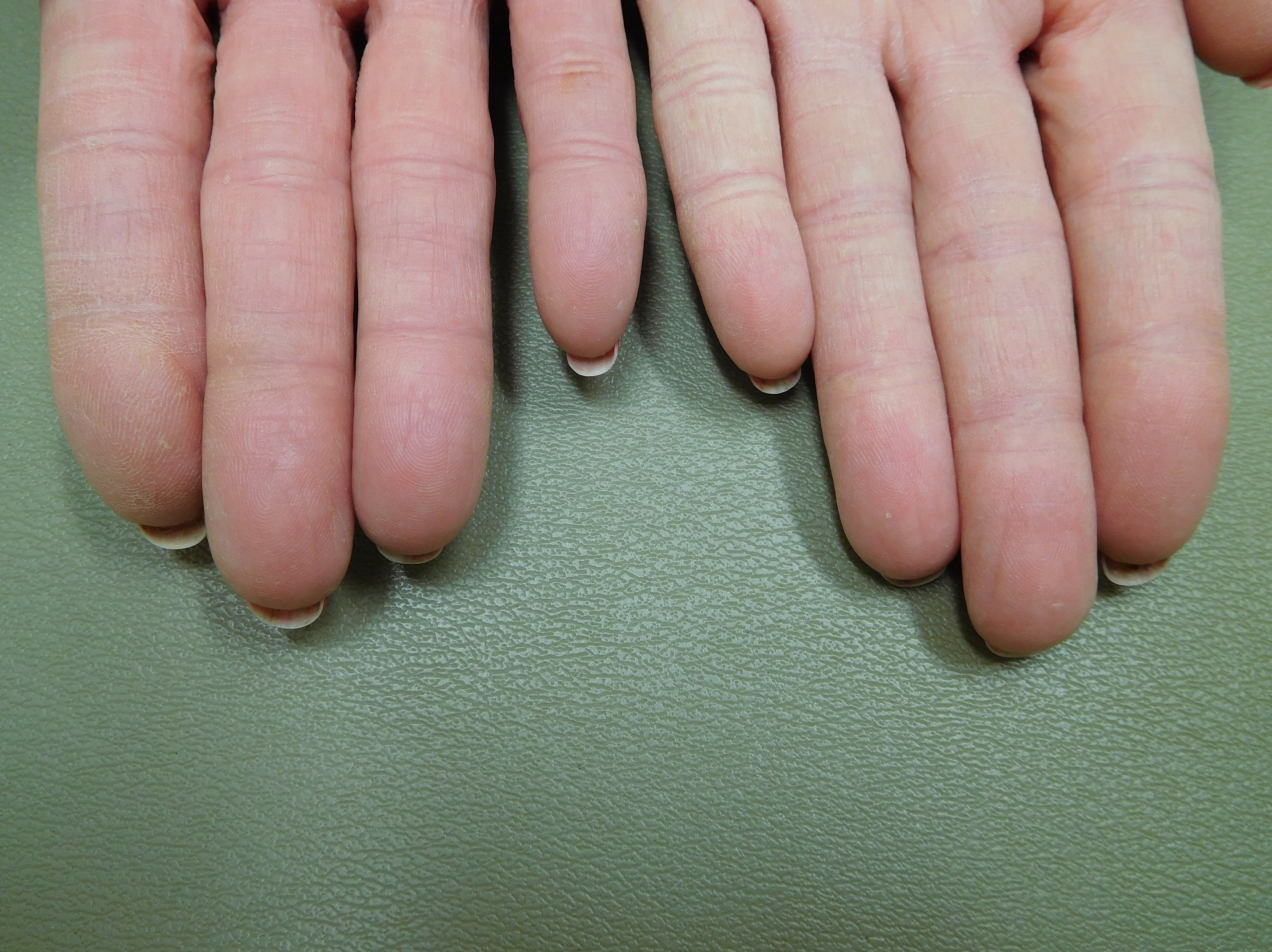
Capecitabine-associated loss of dermatoglyphics on the fingertips of a 73-year-old woman with metastatic breast cancer. More than two years after stopping capecitabine treatment, the palmar surface of her fingertips is erythematous and rough. She is still not able to confirm her identity by fingerprint scanning with her laptop computer.

**Figure 8:**
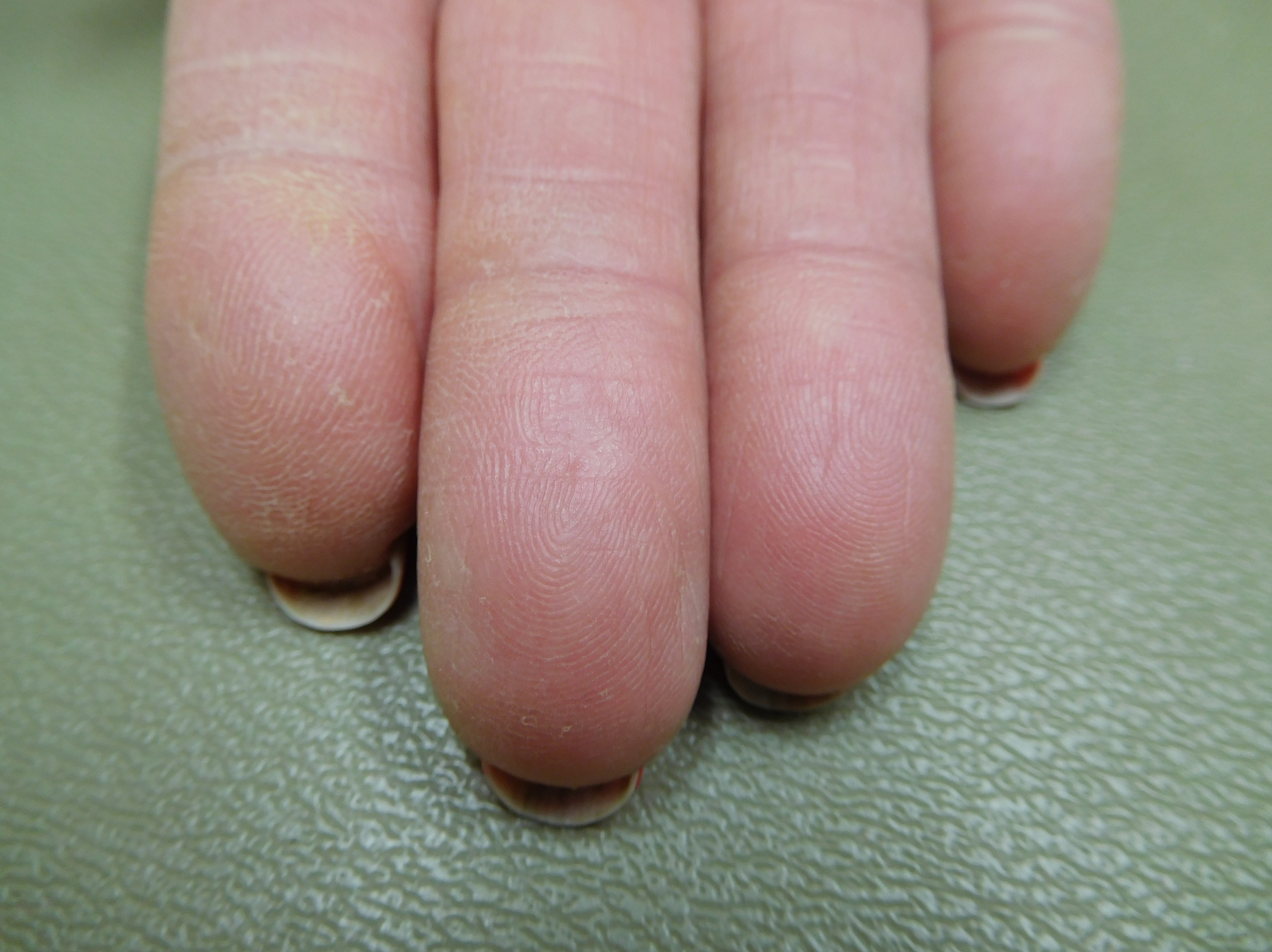
Capecitabine-induced loss of fingerprints on the right hand fingertips of a 73-year-old woman with metastatic breast cancer. Closer examination of the red fingertips shows partial to complete presence of the fingerprint ridges. However, the normal clinical appearance of her fingertips has not returned; pitting and desquamation, with focal longitudinal creases, are also present.

**Figure 9:**
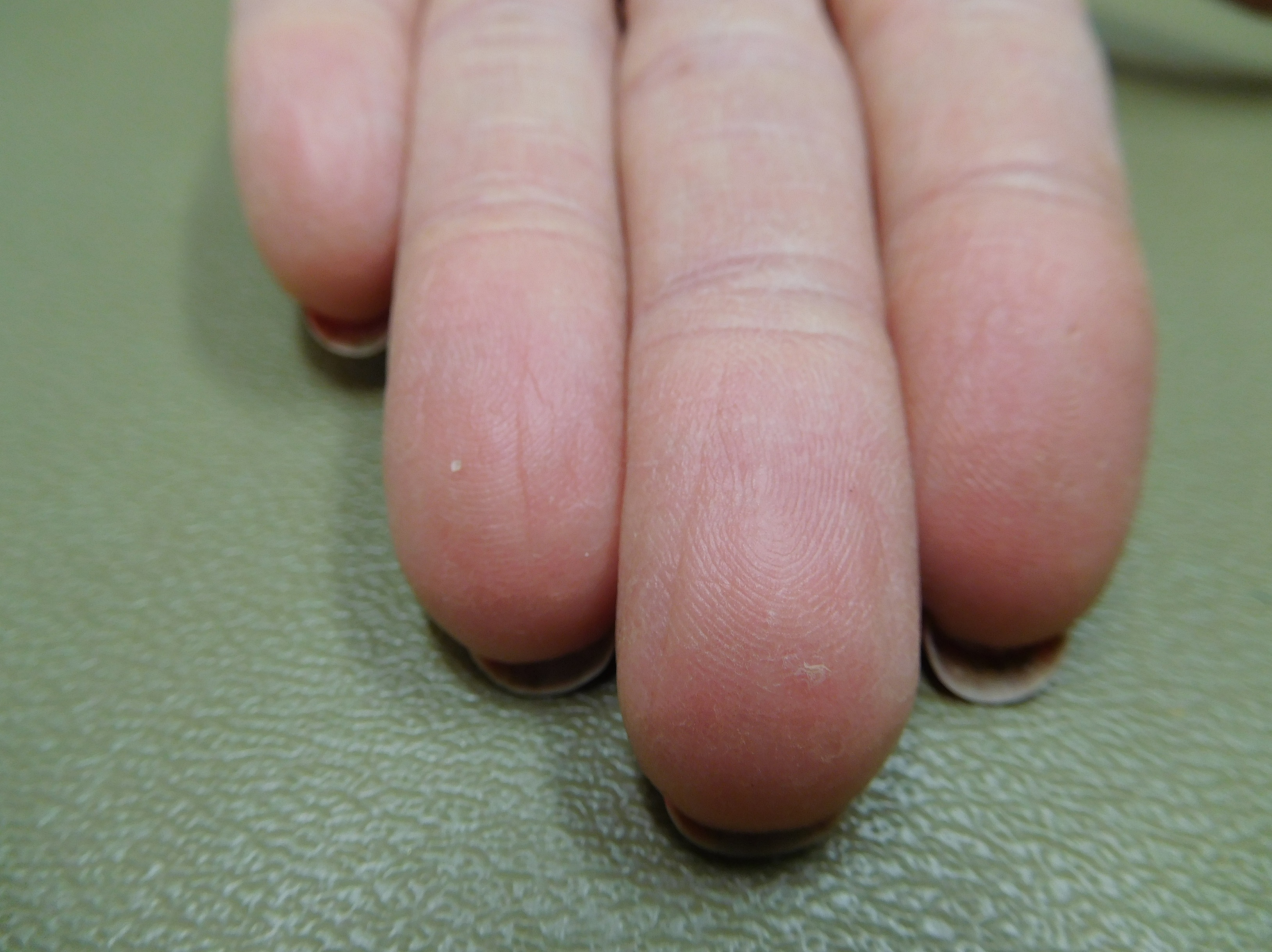
Capecitabine-induced loss of fingerprints on the left hand fingertips of a 73-year-old woman with metastatic breast cancer. Closer examination of the red fingertips shows partial to complete presence of the fingerprint ridges. However, the normal clinical appearance of her fingertips has not returned; pitting and desquamation, with focal longitudinal creases, are also present.

**Figure 10:**
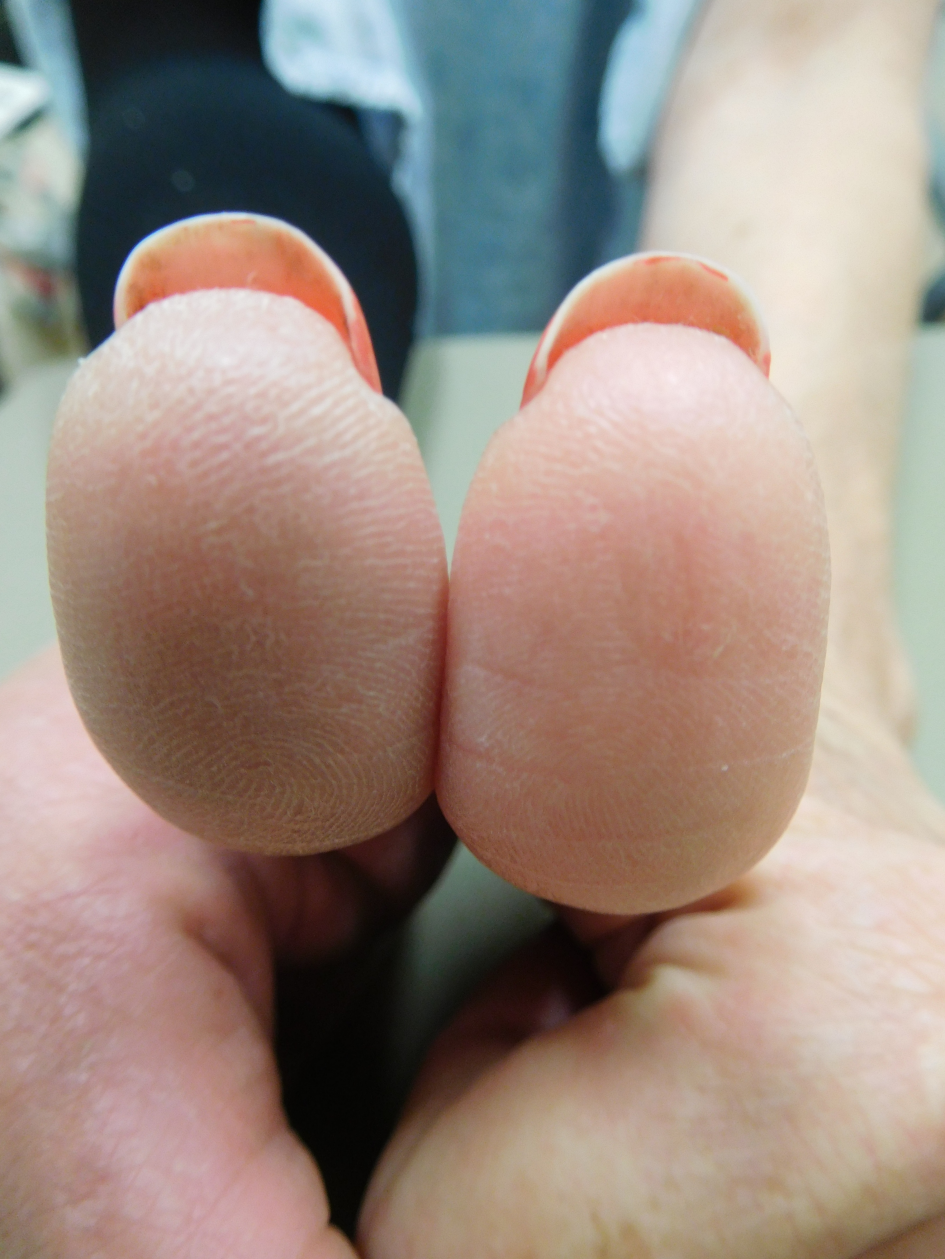
Capecitabine-associated loss of dermatoglyphics on the thumbs of a 73-year-old woman with metastatic breast cancer. Closer examination of the ventral surface of the thumbs shows focal absence of the fingerprint ridges in the central areas. In addition, horizontal creases and desquamation are present.

Examination of her hands in November 2016, more than two years after discontinuing capecitabine, showed that the palmar surface of her fingertips (Figures 7-9) and thumbs (Figure 10) was red and rough. A closer inspection of her erythematous fingertips showed partial to complete presence of the fingerprint ridges; several areas of pitting and desquamation with focal longitudinal creases were also present (Figures 8-9). Although the loss of her fingerprints morphologically appears to have—in part or in total—reversed, she is still not able to confirm her identity by fingerprint scanning with her laptop computer.

## Discussion

Dermatoglyphics (from Greek, 'derma' meaning skin and 'glyphe' meaning carve) are important in anthropology, criminology, medical research, and security [1,3-5]. Fingerprints are used by the immigration services to monitor entrance of foreign visitors into the United States; indeed, difficulties for a patient with congenital adermatoglyphia prompted the condition to be referred to as immigration delay disease [4-6]. More recently, with advances in technology, fingerprint use for personal identification has become integrated for use in banking transactions [7], receiving driver’s licenses [8], obtaining government documents [9], confirming membership to enter athletic facilities, and obtaining access to an individual’s personal computer or telephone.

In a retrospective study, 0.18% of individuals (259 of 145,660 Lebanese persons presenting for new identity pieces) failed to have fingerprints; this corresponds to one of every 563 people. The absence of fingerprints can be congenital or acquired. In addition, the etiologies for acquired adermatoglyphia can be divided into dermatologic (such as dermatitis, hyperhidrosis, psoriasis, and persistent use of potent topical corticosteroids) and non-dermatologic (such as burns, trauma, amputation, degeneration, aging, and idiopathic) etiologies [3].

Capecitabine is an oral antineoplastic agent approved for the management of colorectal and breast cancer [2,4-5,7-10]. Common drug-associated side effects include diarrhea, nausea, vomiting, and stomatitis [2]. In addition, the hand-foot syndrome has frequently been observed [2,5].

Hand-foot syndrome was originally described by Zuehlke in 2004 as an erythematous eruption of the palms and soles in a patient receiving mitotane. It has subsequently been referred to by several synonyms: acral erythema, Burgdorf reaction, palmar-plantar erythema, and plantar erythrodysesthesia. Symptoms and clinical features diffusely affect the palms and soles, varying from minimal erythema to numbness, tingling, burning and pain with edema, desquamation, and/or blisters [2].

It is important to differentiate hand-foot syndrome from hand-foot skin reaction. The latter is most commonly observed in oncology patients receiving tyrosine kinase inhibitors. These individuals develop sharply demarcated painful erythematous and edematous blisters on their palms and soles that evolve into inflamed and tender, hyperkeratotic calluses that may peel; the lesions typically affect the digits and skin sites exposed to pressure with sparing of the central areas of the hands and feet [10].

The National Cancer Institute has three grades of severity for hand-foot syndrome: grade 1, grade 2, and grade 3. There is dermatitis reflected by erythema and peeling or only minimal skin changes in grade 1 hand-foot syndrome; in grade 2, moderate skin changes of peeling, blisters, and edema are present. Ulcerative dermatitis or severe skin changes evident of tissue breakdown, such as bleeding, blisters, edema, and peeling are observed in grade 3 hand-foot syndrome. The hand-foot syndrome is accompanied by altered sensation such as numbness, tingling, and/or burning (grade 1), pain (grade 2) or severe pain (grade 3). For patients with the hand-foot syndrome, the skin changes either do not interfere (grade 1), interfere little (grade 2) or interfere (grade 3) with activities of daily living [2,9]. In addition to the women in this report, it is assumed (but cannot be confirmed) that the severity of hand-foot syndrome for most of the patients with capecitabine-associated adermatoglyphia was evaluated using the National Cancer Institute grading classification [9].

In contrast, the World Health Organization (WHO) has four grades of severity for the hand-foot syndrome that reflect the symptoms, clinical appearance, and pathology of the condition. The severity of symptoms ranges from dysesthesia and paresthesia with tingling of the hands and feet (grade 1) to painless swelling or erythema with discomfort in holding objects and upon walking (grade 2) to periungual erythema and swelling with painful erythema and swelling of the palms and soles (grade 3) to severe pain with desquamation, ulceration, and blistering (grade 4). The clinical appearance of the lesion varies from erythema (grade 1) to edema (grade 2) to fissures (grade 3) to blisters (grade 4). The pathology, if the lesions are biopsied, ranges from dilated blood vessels in the superficial dermis (grade 1) to isolated necrotic keratinocytes in the upper layers of the epidermis (grade 3) to complete necrosis of the epidermis (grade 4). At least one group of investigators used the WHO grading classification to evaluate the severity of hand-foot syndrome for the patient with capecitabine-associated loss of fingerprints that they reported [8].

Capecitabine-induced hand-foot syndrome has been observed in over 50% of patients treated with the drug [2,5]. Possible pathways of capecitabine-related hand-foot syndrome include: cyclooxygenase inflammatory-type reaction, accumulation of capecitabine and its metabolites, enzymes involved in the metabolism of capecitabine, and transporters involved in the absorption of capecitabine [2]. This adverse side effect was noted to be severe in 17% of patients, requiring either dose reduction or discontinuation of the drug [2,5].

Recently, capecitabine-associated loss of fingerprints has been observed in oncology patients who have experienced drug-related hand-foot syndrome (Table 1) [3-10], [current report]. The first patient was described by Wong, et al. in 2009: a 62-year-old man with metastatic nasopharyngeal carcinoma, who received more than three years of capecitabine treatment, was detained at the United States airport customs because the immigration officers could not detect his fingerprints [3]. The next report was published in 2012; Al-Ahwal described a 55-year-old man with metastatic adenocarcinoma of the rectum who received six cycles of combination chemotherapy with capecitabine and oxaliplatin; he was unable to process required government documents during his treatment because of a lack of fingerprints [9]. Subsequently, only six additional papers have been published in either 2015 [3,7-8] or 2016 [4-5,10].

**Table 1:** Characteristics of patients with capecitabine-induced adermatoglyphia a, b, c. a.Abbreviations: A: age (years); Adeno: adenocarcinoma; BT: bank transaction; C: case; Cap: capecitabine; CP: clinical presentation; CR: current report; CuS: customs service detention; DD: dose decreased (of capecitabine); DL: driver’s license; DT: delayed treatment (of capecitabine); FIS: fingerprint identification scanner; G: gender; GDP: government document processing; Grd: grade; HER2: human epidermal growth factor receptor type 2; HFS: hand-foot syndrome; ID: invasive ductal carcinoma; ImS: immigration service detention; LOF: loss of fingerprints; mg: milligram; mg/m: milligram per meter squared; LT: laptop; Nasop: nasopharygeal carcinoma; NS: not stated; On: onset (months after starting capecitabine); PTx: prior antineoplastic treatment; Rec: recovery; Ref: reference; ST: stop treatment (of capecitabine); St4: stage IV; T-: triple negative (lack of expression or estrogen receptor, progesterone receptor and HER2); TA: topical agents; Tx: treatment; USA: United States airport; USB: United States border; W: woman; x: for; -: negative; >: greater than; <: less than. b.Cases 9-12:  In a national survey of fingertips absence of 145,600 Lebanese persons who presented for new identity papers, 259 (0.18%) failed to have fingerprints. Three of the 259 cases of adermatoglyphia were associated with chemotherapy: all of the patients had capecitabine with acral erythema [3]. c.Cases 13-20: A prospective cohort study of capecitabine and the risk of fingerprint loss was performed. Within eight weeks of treatment with capecitabine, severe loss of fingerprints was noticed in nine of 66 (14%) patients with colorectal cancer. Hand-foot syndrome was observed in 46 of 66 (70%) patients treated with capecitabine; the grades for hand-foot syndrome were not associated with the incidence of severe fingerprint quality loss. There was complete recovery of fingerprint quality in either two or three patients; there was no follow-up for six or seven patients [10]. d.She received eight cycles of treatment: 14 days of 1650 mg twice daily followed by seven days off. e.The diagnosis of her recurrent cancer to the right axilla was revised to an apocrine carcinoma; however, multiple biopsies of the bone metastases demonstrated triple negative adenocarcinoma. f.She received 2½ years of treatment: seven days of 2000 mg each morning and 1500 mg each evening followed by seven days off. g.She had partial to near complete reappearance of the fingerprints on her thumbs and fingertips; however, she had not recovered the functional quality of her dermatoglyphics more than two years after discontinuing capecitabine. h.His initial therapy comprised oral capecitabine (1000 mg/m2 twice daily) on days 1-15 followed by a one-week rest, with intravenous oxaliplatin (130 mg/m2) on day 1. After six cycles, his treatment changed to three-week cycles of capecitabine (825 mg/m2) on days 1-14 with bevacizumab (7.5 mg/kg) and irinotecan (220 mg/m2) on day 1; he only received one cycle. i.He received 3½ years of treatment: 14 days of 1750 mg twice daily followed by seven days off.

C	A G	Cancer	PTx	Cap Daily Dose	HFS Grd	HFS Tx	LOF On	LOF CP	LOF Rec	Ref
1	39 W	Breast HER2-	NS	NS	2	DD	NS	USA CuS	No	4
2	57 W	Breast ID,T-	Yes	3300 mg^d^	1	None	0.5	FIS: Gym, Phone	No	CR C1
3	65 W	Breast St4,T-	No	NS	3	TA, DT,ST	3	BT denied	NS	7
4	69 W	Breast ID^e^,T-	Yes	3500 mg^f^	1	None	<1	FIS: LT computer	No^g^	CR C2
5	47 M	Rectal	Yes	2000 m/m x8 months	4	DD,DT	14	DL denied	No	8
6	55 M	Rectal Adeno	No	2000 m/m 825 m/m^h^	3	DT DD,ST	4	GDP denied	NS	9
7	60 M	Rectal Adeno	Yes	NS	2	DD	>6	USB ImS	No	5
8	62 M	Nasop	Yes	3500 mg^i^	2	None	42	USA CuS	NS	6

To date, including the women in this report, capecitabine-related acquired adermatoglyphia has been described in 20 patients. These included four women [4,7], [current report] and four men [3,5,8-9]; the gender was not described for 12 patients [3,10]. They ranged in age from 39 to 69 years; the median age of onset was 59 years.

The two women in this report are Caucasian; the race was not provided for any of the other patients. However, capecitabine-associated loss of fingerprints is a worldwide phenomenon. The investigators for the previously described patients are located in Brazil [8], France [5], Lebanon [3], Mexico [7], the Netherlands [10], Saudi Arabia [9], Singapore [6], and Spain [4]; this is the first report of capecitabine-induced acquired adermatoglyphia in oncology patients from the United States.

All but one of the oncology patients had metastatic disease. Colorectal carcinoma was the most common tumor (12 patients) [5,8-10]. Breast cancer (four patients) [4,7], [current report] and nasopharyngeal carcinoma (one patient) [6] were also associated primary neoplasms. The primary tumor was not described in three patients [10].

Six of the patients had received other antineoplastic therapies prior to treatment with capecitabine [4-6,8], [current report]. Combination therapy with either bevacizumab [7] or oxaliplatin [9] and capecitabine was the initial treatment for two patients. Whether any other agent or treatment modalities were given prior to capecitabine therapy was not described for the remainder of the patients [3,10].

The dose of capecitabine ranged from 825 mg/m2 once daily [9] to 1000 mg/m2 twice daily [8-9]. Some of the patients were receiving either 3300 mg per day or 3500 mg per day divided into morning and evening doses [6], [current report]. The dose level was not stated in 15 of the patients [3-5,10].

The onset of adermatoglyphia was always associated with the patient developing the hand-foot syndrome. However, the severity of hand-foot syndrome varied from grade 1 or grade 2 to grade 3 or grade 4. Indeed, the investigators of a recent study of fingerprint loss in patients treated with capecitabine noted that the incidence of severe fingerprint quality loss was not related to the grade of hand-foot syndrome [10].

The awareness by the patient regarding when their fingerprints had been lost varied. It was noted as early as the patient’s initial capecitabine treatment cycle, as demonstrated by the 54-year-old woman in this report who could no longer be identified by the fingerprint identification scanners of her gym or smartphone. A patient with rectal carcinoma had completed eight months of capecitabine therapy and the drug had been discontinued; he realized the problem half a year later when he was not allowed to receive a driver’s license because he lacked fingerprints [8]. Another man became aware of his absent dermatoglyphics after he had been receiving capecitabine for 3½ years as maintenance treatment for nasopharyngeal carcinoma; he was detained when he traveled to visit relatives in the United States because the immigration officers could not detect his fingerprints [6].

In some of the individuals, the observation of absent fingerprints was an unexpected discovery at the United States airport immigration or border customs checkpoint [4-6]. In addition to the foreign citizens who experienced difficulties when trying to enter the United States, other patients were unable to navigate tasks monitored by their respective governments such as completing documents [9], obtaining a driver’s license [8], or performing a bank transaction [7]. Some of the patients discovered the absence of fingerprints when attempting to use their devices of advanced technology, such as laptop computers or smartphones; they were not being able to gain access using their index finger for identification. One woman could not enter her fitness center—a facility that required confirmation of identity by scanning the member’s fingerprint.

The duration of fingerprint loss is variable. A recent study commented that the loss of fingerprints was reversible in two to four weeks after discontinuation of capecitabine in either two or three of the nine patients who experienced this problem [10]. However, the older woman described in this report, in whom there are clinical features consistent with the partial to nearly complete reappearance of the ridges on her thumbs and fingertips, still did not have functional fingerprints more than two years after discontinuing treatment. In addition, when follow-up of other individuals was described after stopping capecitabine, the loss of fingerprints persisted in three additional patients [4-5,8].

The observation of acquired adermatoglyphia in oncology patients treated with capecitabine is a remarkable—albeit not universal—occurrence. The lack of correlation with the grade of severity of hand-foot syndrome prompted investigators to consider this chemotherapy-induced loss of fingerprints to be a unique adverse event associated specifically with capecitabine [10]. However, it is possible that other antineoplastic agents previously associated with the hand-foot syndrome may have resulted in fingerprint loss, but the newer advances in identification technology were not as widely utilized to enable recognition of the functional loss of the patient’s dermatoglyphics. In addition, loss of fingerprint quality has been identified in a patient who developed drug-induced hand-foot skin reaction while being treated with the tyrosine kinase inhibitor sunitinib [10].

## Conclusions

Capecitabine, an oral 5-fluorouracil prodrug, is currently used in the treatment of metastatic colorectal carcinoma and breast cancer. A frequent adverse event in individuals receiving capecitabine is hand-foot syndrome. Another drug-associated cutaneous side effect, albeit less frequently described, is the loss of fingerprints. Including the two women in this report, capecitabine-induced adermatoglyphia has been described in 20 oncology patients with either colorectal carcinoma (12 patients), breast cancer (four patients), or nasopharyngeal carcinoma (one patient); the primary tumor was not reported in three patients. The capecitabine daily dose varied and was usually divided for administration in the morning and the evening; most of the patients received a total daily dose of either 2000 mg/m2 or 3500 mg. The hand-foot syndrome always preceded the onset of fingerprint loss; however, the severity of the hand-foot syndrome varied from grade 1 to grade 4. In addition, the discovery of capecitabine-associated adermatoglyphia varied from as early as two weeks to as late as 3½ years after beginning treatment. Most of the patients were unaware of their fingerprint loss until they either experienced delays attempting to enter the United States, were unable to process government documents or obtain a driver’s license, or could not obtain access to their telephone, computer, or gym which required fingerprint identification scanning. Although the loss of fingerprints was reversible for some of the patients, several of the individuals did not recover their dermatoglyphics, the functional quality of their fingerprints, or both after discontinuing the drug. The significance of capecitabine-induced adermatoglyphia will continue to increase as fingerprint identification continues to advance not only in scanning technology but also in global utilization. Therefore, it is essential that patients receiving capecitabine are aware of this potential adverse cutaneous sequellae.

## References

[1] Miller JR (1973). Dermatoglyphics. J Invest Dermatol.

[2] Lou Y, Wang Q, Zheng J, Hu H, Liu L, Hong D, Zeng S (2016). Possible pathways of capecitabine-induced hand-foot syndrome. Chem Res Toxicol.

[3] Haber R, Helou J, Korkomaz J, Habre M, Ghanem A, Tomb R (2015). Absence of fingertips with focus on dermatological etiologies: national survey and review. J Clin Dermatol.

[4] Garcia-Saenz JA, Martin M, Diaz-Rubio E (2007). Elementary, my dear Watson. J Clin Oncol.

[5] Mazza C, Slimano F, Visseaux L, Ordan MA, Botsen D, Grange F, Bouche O (2016). Capecitabine and adermatoglyphia: trouble in border!. J Eur Acad Dermatol Venereol.

[6] Wong M, Choo SP, Tan EH (2009). Travel warning with capecitabine. Ann Oncol.

[7] Chavarri-Guerra Y, Soto-Perez-de-Celis E (2015). Images in clinical medicine. Loss of fingerprints. N Engl J Med.

[8] Rovere RK, de Lima AS (2015). Forbidden to drive—a new chemotherapy side effect. Klin Onkol.

[9] Al-Ahwal MS (2012). Chemotherapy and fingerprint loss: beyond cosmetic. Oncologist.

[10] van Doorn L, Veelenturf S, Binkhortst L, Bins S, Mathijssen R (2017). Capecitabine and the risk of fingerprint loss. JAMA Oncol.

